# Wave‐Partition‐Governed Dual‐Site Spallation in Single Crystals

**DOI:** 10.1002/advs.202515623

**Published:** 2025-12-08

**Authors:** Youlin Zhu, Sheng Qian, Lianfu Qiu, Jianian Hu, Guoqiang Luo, Qiang Shen, Qi Tong

**Affiliations:** ^1^ Department of Aeronautics and Astronautics & College of Intelligent Robotics and Advanced Manufacturing Fudan University Shanghai 200433 China; ^2^ State Key Laboratory of Precision Blasting Jianghan University Wuhan 430100 China; ^3^ State Key Lab of Advanced Technology for Materials Synthesis and Processing Wuhan University of Technology Wuhan 430100 China

**Keywords:** anisotropy, molecular dynamics, shock loading, spall

## Abstract

The spall failure in shock‐loaded perfect single crystals reveals significant orientation dependent characteristics, yet the fundamental relationship between shock wave propagation anisotropy and fracture mechanisms remains unclear. Employing large‐scale molecular dynamics simulations, a novel dual‐spallation phenomenon governed by anisotropic elastic–plastic wave separation is discovered. Crystal orientations exhibiting stronger wave separation (e.g., [111]) demonstrate a characteristic two‐stage spallation pattern, contrasting sharply with the conventional single‐spallation behavior observed in orientations like [100]. Systematic analysis through quantitative void distribution statistics, fracture surface energy evaluation, and modified Nucleation and Growth (MNAG) model reveals that this wave‐mediated mechanism improves damage resistance through two synergistic effects: attenuation of rarefaction wave interaction and suppression of catastrophic void coalescence. The findings establish stress wave engineering via crystalline orientation control as a vital strategy for developing next‐generation impact‐resistant materials, providing both fundamental insights into dynamic fracture physics and practical guidelines for material design.

## Graphical abstract

Crystalline anisotropy shapes shock‐wave propagation and the resulting damage evolution in single crystals. Large‐scale molecular dynamics and damage modeling uncover a dual‐spallation mechanism driven by anisotropic elasticplastic wave separation, which mitigates damage accumulation. This mechanism offers a promising route for designing extreme‐environment nanomaterials.

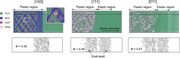

## 1 Introduction

The dynamic fracture behavior of metallic materials under extreme loading conditions constitutes a fundamental research focus with critical implications for impact mitigation and structural safety applications.^[^ ^1^, ^2^ ^]^ Among various failure modes, spallation, arising from uniaxial tensile stresses generated through rarefaction wave interactions, features in high‐strain‐rate shock scenarios. At the microscale, this ductile failure process progresses through three distinct stages: void nucleation, growth, and coalescence, with kinetics modulated by coupled thermal‐mechanical effects, shock intensity, and inherent material heterogeneities. Studying spallation can be challaging since it's highly related with preceding behaviors such as melting, phase transition, and plastic deformation.^[^ ^3^, ^4^, ^5^, ^6^, ^7^ ^]^

Starting with simple face‐centered cubic (FCC) single crystals, the anisotropic nature of spallation has attracted considerable attention. While quasi‐static tensile tests predict superior failure resistance along the [111] orientation based on analysis of Schmid factor (0.272 vs 0.408 for [100]) and activated slip systems activation, shock loading experiments reveal contradictory trends across different FCC systems. Early plate‐impact studies on aluminum by Chen et al.^[^ ^8^ ^]^ demonstrated higher spall strength in [100] orientations (6–45 GPa range), initially attributed to crystalline purity effects. Subsequent investigations by Owen et al.^[^ ^9^ ^]^ revealed an inverse strength hierarchy ([111] > [011] > [100]) at lower stresses (4–10 GPa), aligning with quasi‐static predictions through Hugoniot Elastic Limit (HEL) measurements. Recent work by Millett et al.^[^ ^10^ ^]^ reinforced this orientation dependence via Schmid factor arguments, yet such trends prove material‐specific, materials with low stacking fault energy like copper or tantalum^[^ ^11^ ^]^ consistently exhibit maximum spall strength in [100] orientations, as shown by Turley et al.'s velocity profile analyses, where [100] specimens displayed superior strength however, accelerated void growth rate.^[^ ^12^ ^]^

This persistent controversy in orientation‐dependent spallation behavior stems from two fundamental experimental limitations: post‐mortem characterization cannot capture real‐time damage evolution, and conventional diagnostics overlook the intrinsic anisotropy of shock wave propagation. Specially, under weak shock conditions, the temporal separation between elastic precursors and plastic waves create distinct stress states that may precondition material damage.^[^ ^13^, ^14^, ^15^, ^16^, ^17^ ^]^ While macroscopically dismissed due to fast HEL attenuation and plastic wave chasing in sustained loading, recent advances in multiscale study, reveal persistent elastic precursor effects under extreme strain rates.^[^ ^18^ ^]^ Molecular dynamics (MD) studies also demonstrate that these ultrafast conditions preserve high‐pressure elastic precursor,^[^ ^11^, ^19^, ^20^, ^21^ ^]^ potentially enabling novel failure pathways. Nevertheless, current understanding remains fragmented regarding how two‐wave separation modulates spallation processes across different crystallographic orientations.

Herein, we use non‐equilibrium molecular dynamics (NEMD) simulations^[^ ^22^, ^23^, ^24^ ^]^ encompassing eight representative FCC orientations (For more ore details one can refer to the Method part and supporting information). Our investigation focuses on how wave structure dictates damage localization patterns, void evolution, and ultimate fracture toughness. By combining spatiotemporal damage analysis with wave propagation characterization, we identify a previously unreported dual‐spallation governed by anisotropic elastic–plastic wave separation.

## 2 Results and Discussion

### 2.1 Elastic–Plastic Wave Separation

**Figure 1:**
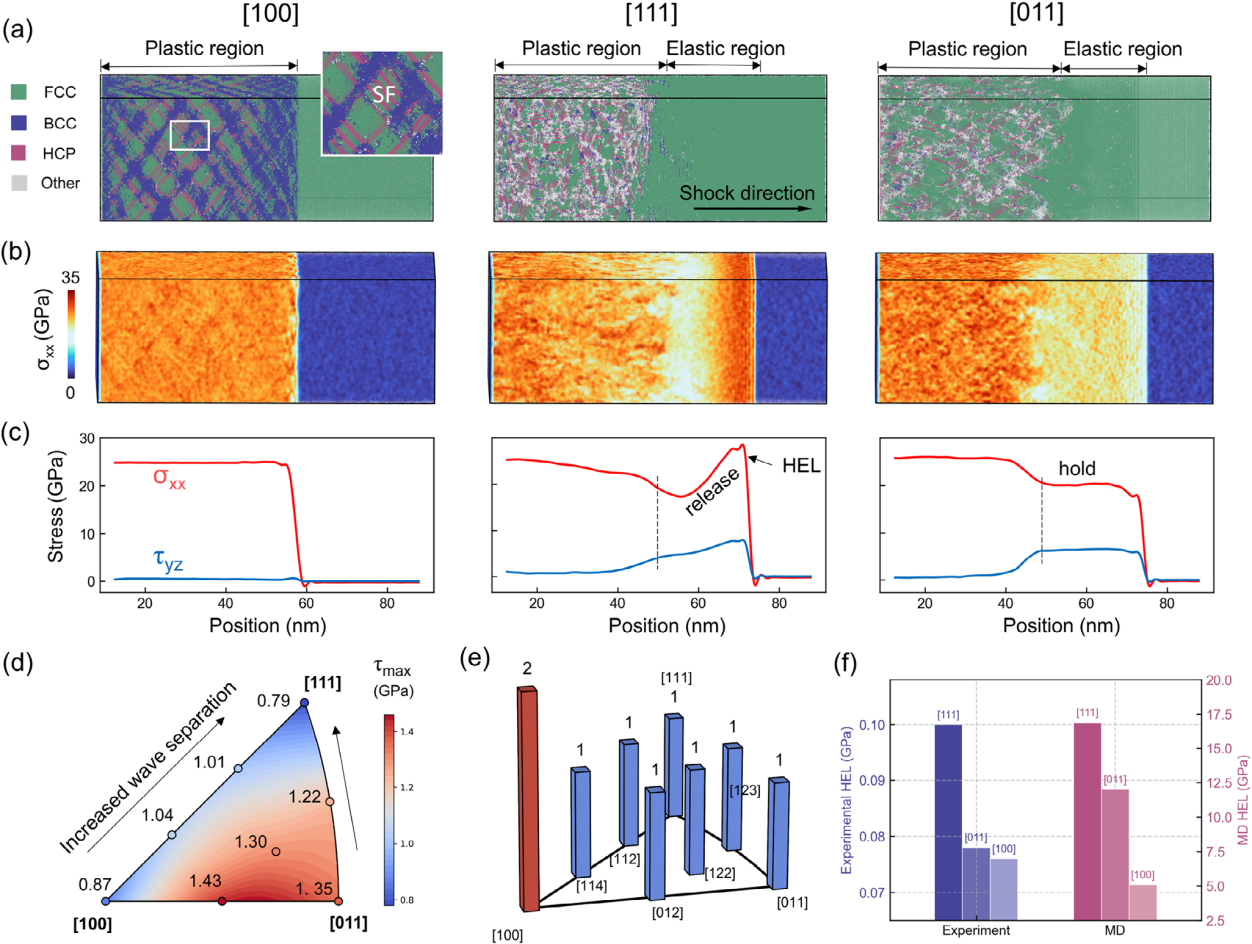
Elastic–Plastic Wave separation. a) Atomic snapshots of single‐crystal Al in 3 main orientations under shock compression at *t* = 8 ps. b) Cloud maps of compressive stress distributions. c) Compressive and shear stress wave profiles. d) Maximum resolved shear stresses and e) number of initiable slip systems for 8 orientations at a compression ratio of 0.95.f) Comparison of HEL value in MD simulation and shock experiment of single crystal Al by Owen.^[^ ^6^ ^]^

By piston method, we implement a 1.3 km s^−1^ shock for eight uniformly distributed orientations from the inverse pole figure for systematic investigation of damage distribution, with three primary orientations chosen for in‐depth analysis of a shock wave structure. The anisotropic shock wave propagation in single‐crystal aluminum generates distinct stress wave signatures (**Figure** **1**b,c): the [100] shock orientation demonstrates a monolithic plastic wave profile, contrasting sharply with the elastic–plastic dual‐wave separation observed in [111] and [011]. Post‐wave characteristics further differentiate these orientations ‐ [111] exhibits rapid stress attenuation while [011] sustains precursor plateaus. Atomic snapshot (Figure 1a) reveals dislocation‐free elastic regions despite extreme pressures (20–25 GPa), confirming purely elastic compression. In the [100] orientation, plastic deformation is accommodated by the nucleation and glide of Shockley partial dislocations on the primary {111} slip planes.^[^ ^25^ ^]^ This results in the formation of stacking faults, which are inclined at ≈45° relative to the loading direction. At the intersections of these stacking faults, severe lattice distortion and complex dislocation reactions occur, leading to local regions with non‐FCC coordination^[^ ^20^ ^]^ (see subplot in Figure 1a). This mechanism effectively mitigates shear stress accumulation, resulting in a nearly constant shear stress profile for [100] with minimal fluctuation. In contrast, the [111] and [011] orientations exhibit significant post‐wave generation of disordered atoms and complex dislocation structures.

A direct comparison between macroscopic experiments and atomic simulations is challenging. However, some evidence can still be found in terms of wave profiles. For instance, in the plate impact experiments on single‐crystal aluminum (see Figure 1f) conducted by Owen et al,^[^ ^6^ ^]^ the HEL (Hugoniot Elastic Limit) values calculated from the free surface velocity jumps are 0.104, 0.078, 0.076 GPa in [111],[110] and [100], respectively, following a trend [111] > [110] > [100], which is consistent with our simulation results (shown in Figure 1f) and can be well explained in the following paragraph. Still, atomic simulation result observed here do diverge from macroscopic observations,^[^ ^26^ ^]^ on the one hand, compared with MD results, HEL values are usually mild in experiments, this is because defects due to materials fabrication can lead to much lower HEL values. On the other hand, some studies^[^ ^27^, ^28^, ^29^ ^]^ have reported the existence of elastic precursor waves along the [100] crystallographic direction (which is absent in this work. HEL of MD [100] in Figure 1f was approximated from 20% of pressure^[^ ^12^, ^13^ ^]^). While the validity of interatomic potential functions might initially be questioned (comprehensive validations are provided in Supplementary materials), this discrepancy actually stems from the constrained shock propagation distances inherent to molecular dynamics timescales and spatial dimensions. However, this scale‐dependent discrepancy does not compromise the fundamental anisotropy governing the dual‐wave splitting mechanism.

The release of shear stress is the main cause of the separation of the double waves, in face‐centered cubic (FCC) crystals, where plastic deformation predominantly occurs through 1/6<112 > slip systems, we conduct systematic calculations of both the largest resolved shear stresses (τ_*max*_) among 12 slip systems and the corresponding number of activated slip systems under different crystallographic orientations.^[^ ^25^ ^]^ This continuum solid mechanics approach proves more appropriate than Schmid factor analysis for characterizing uniaxial strain conditions in plate impact scenarios.^[^ ^25^ ^]^ As demonstrated in Figure 1, the [111] orientation manifests the smaller τ_*max*_ values, indicating inherent resistance to slip initiation. In addition, the number of initializable slip systems in each orientation is calculated and presented in Figure 1e. Given the universal occurrence of spallation across all specimens, we adopt a criterion wherein secondary slip systems become operational simultaneously upon reaching 70% of τ_*max*_ at 0.95 compression ratio. The [100] orientation uniquely exhibits concurrent activation of two independent slip systems, contrasting with single‐system activation in other orientations. Therefore, a smaller value of either τ_*max*_ or initializable slip systems leads to enhanced barriers for Shockley dislocation nucleation. The combined effect constitutes the progressive increase of the dislocation resistance from [100] through [011] to [111] orientations. This stress dissipation bottleneck consequently amplifies transient elastic–plastic wave separation due to impeded stress relaxation dynamics.

### 2.2 Dual‐Spallation

**Figure 2:**
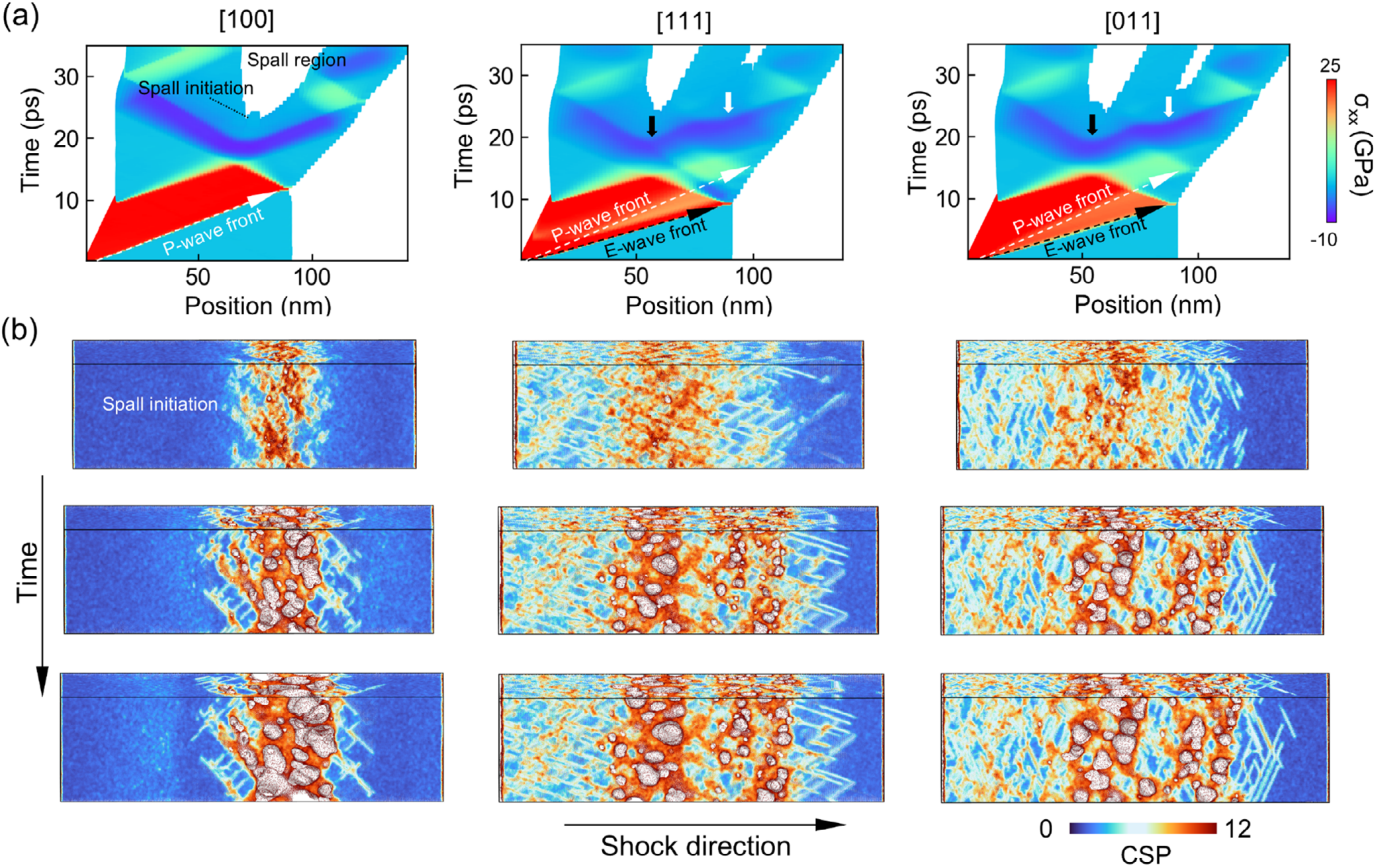
Dual‐spallation phenomenon. a) Position‐Time (X‐T) clouds of wave interaction in three main orientations. The dashed arrows indicate the separations of the elastic (E‐wave, white) and plastic (P‐wave, black) waves. The solid arrows indicate the unloading reflection wave intersections (E‐wave in white and P‐wave in black). b) CSP‐colored atomic snapshots at *t*_0_ (the time of spall initiation), *t*_0_ + 2 ps, and *t*_0_ + 5 ps during the spall processes, with fracture surfaces colored in grey.

**Figure 3:**
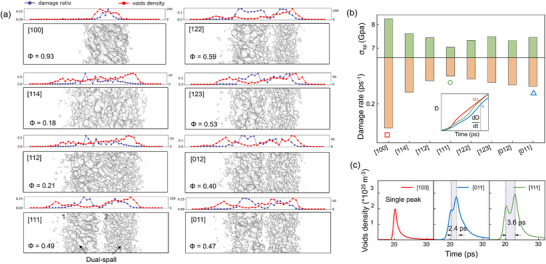
a) Distributions of void surface in various orientations when the damage ratio reaches 0.1. The order parameter Φ characterizes the degree of void coalescence. b) Spall strength and damage accumulation rate of the system in each orientation. The inset presents the temporal evolutions of the void volume fraction *D* in [100], [011], and [111] shock orientations. c) Temporal evolutions of the void number density.

The phenomenon of double‐wave separation results in a more complex wave system upon interaction with the free surface, directly influencing the damage characteristics of the system's spall layers. Through a position‐time cloud we can track the propagation path of the shock wave within the material (see **Figure** **2**a). The separation between elastic waves (E‐wave) and plastic waves (P‐wave) intensifies as the stress wave propagates. Under the same impact velocity, the tensile stresses induced by both impact pressure and rarefaction waves in the [011] and [111] orientations are significantly lower than those in the [100] direction. In the [111] orientation, due to substantial stress relaxation following the elastic wave, the rightward‐propagating plastic wave is strongly influenced by the leftward rarefaction wave, resulting in a high degree of wave separation. Consequently, the onset of secondary spall occurs considerably later compared to other orientations. This pronounced wave separation further leads to a large number of small‐sized voids remaining within 5 ps after spall initiation, as shown in the fracture snapshot in Figure 2b, where atoms are colored by centrosymmetry parameters (CSP). We employ a local averaging algorithm when doing CSP sequential coloring to better highlight surface defects and voids, while may lower clarity of the stacking faults. In the [100] sample, damage is primarily localized at a single site. However, in the other two orientations, the interaction between the elastic precursor wave, the rarefaction wave formed by plastic wave reflection, and the unloading rarefaction wave gives rise to a novel phenomenon of dual‐spallation within the single crystals, see white solid arrow in Figure 2a. The key distinction between dual‐spallation and a wider single spallation lies in the fact that in the dual‐spallation scenario, the elastic precursor wave undergoes a release process due to the thorough separation of elastic–plastic wave (see [111] in Figure 1c). Thus, during the tensile phase of the system, the rarefaction wave from the piston release will intersect sequentially with two relatively independent reflected waves. This causes the left and right parts of the system to undergo independent nucleation processes, respectively. In the [111] orientation, this manifests as a clear dual‐spallation. When the separation between elastic and plastic waves is less pronounced, as observed in the [110] orientation, for instance, the two spallation sites are positioned very close to each other. This scenario can be interpreted as a widened single spallation. The damage snapshots across all eight orientations precisely capture this transition from single to dual spallation. The damage distribution maps at a damage ratio (D, void volume fraction) of 0.1, as shown in **Figure** **3**a, indicate that the increasing degree of double‐wave separation due to anisotropy will cause the spall mode to gradually transit from single to dual. The order parameter Φ (defined as the maximum void volume divided by the total void volume) is labeled. Initially developed by Alejandro^[^ ^30^ ^]^ to characterize critical void behavior during fracture processes, this dimensionless parameter has become an established metric for detecting fracture penetration thresholds. Catastrophic material failure typically occurs when complete cavity interconnection happens, driving the Φ parameter asymptotically toward 1. The fixed damage value of 0.1 is recognized in engineering as the criterion for determining whether complete spallation occurs in Aluminum.^[^ ^31^ ^]^ In our simulations, due to differences in stress wave propagation across different orientations under the same shock intensity, comparing damage at the same time would be biased. For instance, damage in the [100] orientation initiates earlier and develops more rapidly than in the [111] orientation. Using a fixed damage value for comparison allows us to focus more on contrasting the distribution characteristics of damage distribution itself. Specifically, [100] orientation exhibits typical damage, characterized by rapid void coalescence within a narrow region along the impact direction, resulting in an order parameter of 0.93. In contrast, the spall region for the [114] and [112] orientations are significantly wider, but still uniform, corresponding to an order parameter of ≈0.2, with central voids yet to undergo substantial coalescence. For other orientations, such as [111], the pronounced elastic–plastic wave separation during compression leads to greater spacing between the rarefaction wave stretching locations, facilitating dual spallation.

Notably, the damage on the left side of the sample is driven by rarefaction waves reflected from the elastic precursor, resulting in higher void aggregation compared to the spall on the right, which are caused by rarefaction waves reflected from the plastic wave. The voids in the first failure site on the left side nucleate earlier, resulting in larger sizes and lower quantities compared to those in the failure region on the right. One can also observe this in Figure 2a, for the same model and under the same shock intensity, we determined the onset of nucleation by monitoring a fixed‐thickness slice (1.6 nm) within the system. Nucleation was identified when the number of atoms in this slice dropped below a threshold value (N = 50 000). The blank regions in the cloud diagram clearly show that spallation on the left side occurs earlier than on the right. This is attributed to a shorter period for nucleation as well as different motion behavior of dislocations under the action of elastic and plastic rarefaction waves. The order parameter for this spallation mode is ≈0.5, meaning that the largest void accounts for half of the total void volume. Additionally, in the [111] orientation, the voids in the second spall are significantly smaller than those in other orientations. This observation is further supported by the void number density data in Figure 3c, where void evolution in the [100] orientation occurs rapidly, forming narrow peaks that do not undergo splitting. In contrast, the peaks in the [011] and [111] orientations exhibit splitting and progressively broaden over time. Together with an increase of maximum voids density, this indicates a reduction in the interference between the two spallation sites, which, to some extent, confirms the previously mentioned trend of increasingly pronounced double‐wave separation.

### 2.3 Damage Mitigation of Dual‐Spallation

Our further detailed data and analysis have demonstrated that the aforementioned phenomenon is conducive to a significant reduction in the severity of the damage. 3b compares the spall strength and damage rate (defined as Δ*D*/Δ*t* from *t* = *t*_0_ to *t* = *t*_0_ + 5 ps) across various crystallographic orientations. The orientation effect on spall strength (σ_*sp*_) has been widely reported in the literature and is primarily negatively correlated with the density of immobile dislocations, such as stair‐rod and Hirth dislocations. The formation and entanglement of 1/6<112 > dislocations contribute to the anisotropy of spall behavior. Due to the complexity of wave interactions, this study determines spall strength solely through the binning method, namely the ultimate tensile strength. This implies that whether spallation occurs as a single or dual event does not affect its orientation dependence. Consequently, our result on the orientation dependency of spall strength closely aligns with that of Li ’s study^[^ ^25^ ^]^ on single‐crystal Cu, though differ from Al's macroscopic experimental observations ([111] exhibit the highest spall strength), likely due to the extreme strain rates inherent in molecular simulations. What surprises us is that, through Figure 3b, the damage accumulation rate in orientations exhibiting dual spallation is significantly lower than their counterparts in single spall, stronger orientations exhibit faster damage progression. The wave system structure (governed by orientation) and the intrinsic strength of various orientations share a common origin: the ease with which plastic deformation occurs under external loading in a given orientation. Since orientations that resist plastic deformation are also more prone to elastic–plastic wave separation, this inverse relationship ultimately leads to the observed synergetic trends in spall strength and damage rate across various crystallographic orientations. By comparing the characteristic curves of damage evolution over time (see subplot), we reasonably conclude that this counterintuitive phenomenon arises from the double‐wave structure, which reduces void interaction and subsequently hinders void coalescence. Still, it is necessary to exclude the influence of the intrinsic differences in damage accumulation difficulty for spallation across crystal orientations. This ambiguity is further explored through a well‐designed ramp wave loading simulation^[^ ^32^, ^33^ ^]^ as detailed in the Supplementary Material. Under ramp wave loading condition, [111] oriented elastic–plastic wave splitting appeared later (see dash boxed in Figure S3a, Supporting Information), and its subsequent reflected rarefaction wave did not intersect with the rightward unloading rarefaction wave, resulting in the absence of dual‐spall, and a wider single‐point fracture region forms instead, see Figure S3b (Supporting Information). On the other hand, [011] retains its dual‐spall characteristics because of the stable precursor wave platform. Comparing the damage curves, it is found that the loading mode makes the damage regulation reversed, so that the single‐point spall occurs in the [111] orientation under the ramp wave loading, with the damage accumulates fastest, whereas the [100] is slower, and the [011] is faster in the early stage, but slower in the final stage. Taken together, the above results bypass the effect of the intrinsic ease of damage accumulation and confirm that the occurrence of dual‐spalls is the key to mitigate damage accumulation.

With the dual spallation mechanism established, we employ the modified Nucleation and Growth (MNAG) model, combined with Nondominated Sorting Genetic Algorithm II (NSGA2), to accurately reconstruct the dual spallation process and compare it with single spallation. The MNAG model builds upon the widely‐known nucleation‐growth semi‐empirical model (NAG) proposed by Curson et al.,^[^ ^34^, ^35^, ^36^, ^37^, ^38^ ^]^ incorporating a refined void merging term by fitting a polynomial to the stress history as an input for the cavity growth equation.^[^ ^39^ ^]^ A time‐step integration method is then used to obtain the damage evolution history.

**Figure 4:**
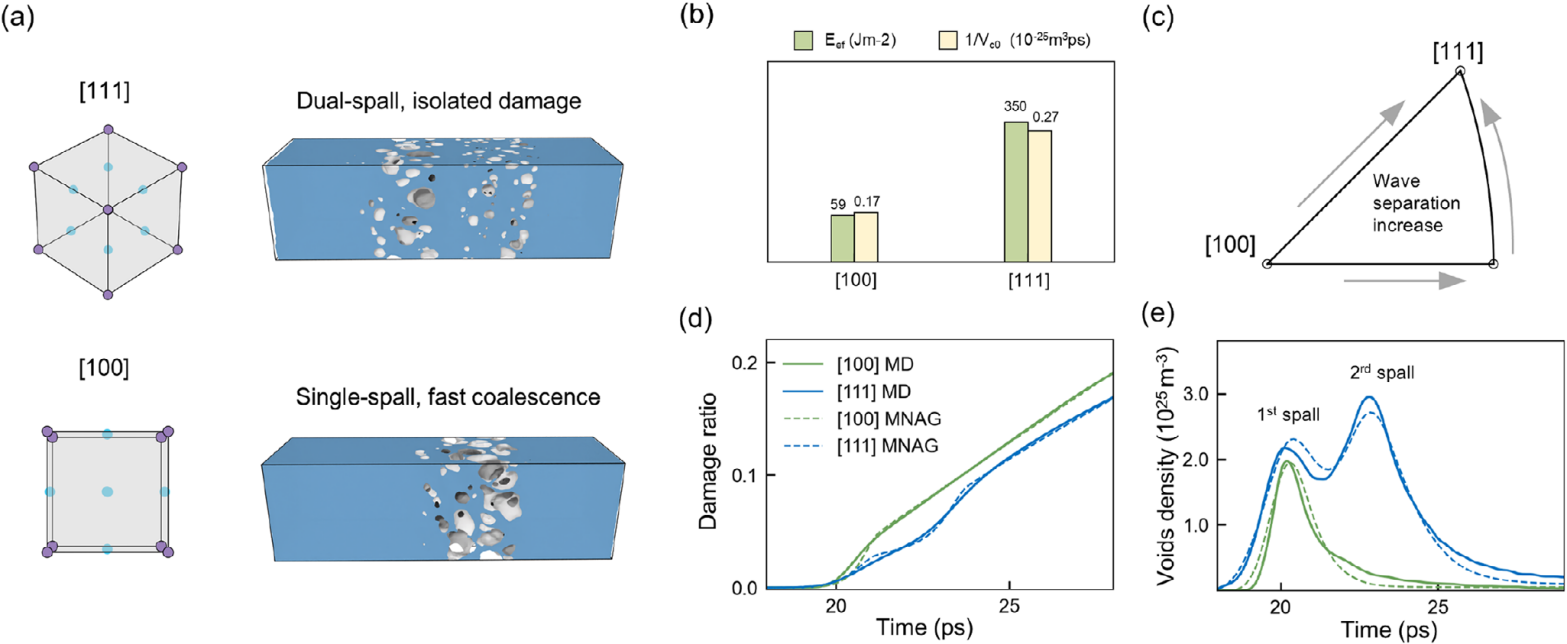
A comparison between single with dual spallation in a) damage features, b) Effective fracture surface energy *E*_*ef*_ and fitted coalescence parameter *V*_*c*0_, d) void number density and e) damage ratio evolution result of MD and MNAG models.

Assuming that the two spallation sites in the [111] orientation are completely independent, the model is then constructed based on the stress histories of both spall regions and their respective onset times. The fitted void number density and damage degree results, shown as dashed lines in **Figure** **4**d,e, exhibit good agreement with the MD data. The primary sources of error stem from the fact that the void growth equations assume strict adherence to exponential growth, which is not fully maintained in the non‐equilibrium state. Computational details about MNAG model are provided in the Supplementary Materials.

In the fitting results, the segmented void merging process in dual spall fracture is evident, with damage ratio elevating and simultaneously, peak density decreasing in 2 stages (see 1st and 2nd spalls labeled in Figure 4e). Dual spallation in the [111] orientation initiates earlier, yet the damage accumulation is significantly slower. The coalescence coefficient in the MNAG model quantifies the contribution of voids merging to damage evolution and numerically determines the slope of the curve in the inertia growing stage (after 25 ps). The fitted merging coefficients *V*_*c*0_ for the [100] and [111] orientations are 5.95 × 10^25^ and 3.73 × 10^25^m^−3^ps^−1^, respectively. Combined with the damage evolution curves in Figure 4b, it is evident that greater double‐wave separation leads to reduced interference between the two fracture sites. Compared to single spallation, the lack of void merging in dual spallation significantly suppresses overall damage accumulation.

This conclusion is further supported by the effective fracture surface energy calculations using the Grady method,^[^ ^40^ ^]^ expressed as *E*_*ef*_ = *YDsN*, where *Y* represents the flow stress of the spall region, *D* is the damage degree (here we choose *D* = 0.1), and *s* denotes the equivalent void size (more details in supplementary materials). *N* is the voids number. As shown in Figure 4b, *E*_*ef*_ in the [111] orientation is three times higher than that in the [100] orientation. Although the factors such as shock wave attenuation and intrinsic heterogeneity are also at play, the comprehensive analysis confirms that the dual spallation phenomenon caused by orientation‐dependent double‐wave separation significantly inhibits spall damage accumulation. Moreover, our simulation results exhibit phenomenological similarity to the macroscopic multiple fracture, recently observed in the aluminum alloy by Li et al in their plate experiment,^[^ ^41^, ^42^ ^]^ although their results were achieved under varying loading conditions.

## 3 Conclusion

In summary, we observed a novel phenomenon of dual spall fracture in single‐crystal Al, driven by elastic–plastic wave segregation during ultrafast impacts, through large‐scale molecular dynamics simulations. By integrating atomic‐scale snapshots, void statistics, and numerical modeling, we demonstrated that this phenomenon effectively slows down damage accumulation under the same shock intensity. The novelty of this work lies in the discovery of the specific phenomenon of dual‐site spallation, which has not been reported previously, either through simulations or experiments. Furthermore, we elucidate the underlying mechanism by which the intrinsic structure influences stress wave propagation during compression and the subsequent fracture process.

While this study focuses on aluminum due to the considerations such as the potential function, we posit that all crystalline materials exhibiting pronounced stress wave propagation anisotropy may demonstrate dual spall fracture behavior. Such phenomena could become universally observable when future diagnostic techniques achieve sufficient spatiotemporal resolution, it may be possible to manipulate stress waves, either diverging or focusing them, by tailoring local crystal orientations, which presents substantial potential for tunable material modulation in practical engineering applications.The results obtained from MD simulations are transferable to broader contexts, as studying the fundamental mechanisms of spallation and fracture at the nanoscale under ultra‐high strain rates is highly valuable for isolating intrinsic physical effects. Furthermore, the strain‐rate regime explored in this study is not purely theoretical. It is also applicable to certain experimental conditions, such as laser‐driven shock experiments, where similarly extreme loading rates (up to 10^9^ *s* ^−1^) can be achieved.^[^ ^43^, ^44^ ^]^ Although the practical application of perfect single crystals faces challenges in preparation and other limitations, our findings offer new insights into stress wave modulation and impact protection, providing a potential avenue for materials design under extreme conditions.

## 4 Experimental Section

Large MD simulations of ≈6 million atoms were performed using LAMMPS with the EAM potential of aluminum,^[^ ^26^ ^]^ the length of boxes in shock direction (x‐) was kept near to 90 with 30nm in y‐ and z‐ direction. Free boundary conditions were applied in x‐direction, while periodic boundary conditions were applied in y‐ and z‐ to reduce the boundary effect. Energy minimization was performed by the conjugate gradient (CG) method to reach a local minimum‐energy state, then, samples are relaxed in an NPT ensemble at 300 K and 0 Pa for 50 ps to enable appropriate atomic relaxations at the grain boundaries. After that, a shock simulation was carried out in an NVE ensemble with a timestep of 1 fs.

A rigid piston method was used to introduce shock wave, a 5 Å‐thick rigid piston on the left side of the target moves along x‐ direction at 1.3 km s^−1^ for a period to bring spall fracture in all sample, meanwhile avoiding intense thermal soften or micro‐spall. Pulse duration holds constant at 10 ps such that spall appears predictably in the middle of the sample. After loading's completion, the rigid piston was removed, and a subsequent rarefaction wave generates and propagates. The atomic configuration was snapshotted with the powerful software Open Visualization Tool (OVITO), Moreover, integrated modules Construct Surface Mesh were used to identify voids or damage accumulations.

Based on the binning analysis, the maximum tensile strength was taken as the spall strength (σ_*sp*_). The sound approximation method was not used to calculate σ_*sp*_ because complex wave interactions arose at various orientations. Tensile stress was calculated from the Virial‐ theorem component on the x‐axis of all atoms. It was noted that the thermal vibration velocity in the x direction within the non‐equilibrium region behind the wavefront cannot be directly obtained. Yet, it was approximated by *v*_*ix*_ = 1/2(*v*_*iy*_ + *v*_*iz*_) due to the isotropy of thermal vibration. Grady's model and MNAG model are used to fit MD result on a multiscale level, more details can be found in the Supporting Information. This study does not involve statistical analysis.

## Conflict of Interest

The authors declare no conflict of interest.

## Supporting information

Supporting Information

Supplemental Movie 1

Supplemental Movie 2

## Data Availability

The data that support the findings of this study are available from the corresponding author upon reasonable request.
