# Supplementary material for: Wave‐Partition‐Governed Dual‐Site Spallation in Single Crystals
Source: Adv Sci (Weinh). 2025 Dec 8;13(9):e15623. doi: 10.1002/advs.202515623 (PMC12904060; doi:10.1002/advs.202515623)
Supplement: Supplementary file 1 — Supporting Information [file ADVS-13-e15623-s002.docx]

**Supplementary Materials**

**Wave-Partition-Governed Dual-Site Spallation in Single Crystals**

1. **Non-equilibrium Molecular dynamics simulation details**

We perform large MD simulations of about 6 million atoms using LAMMPS, with the EAM potential of aluminum developed by Zhahovskiiis^[1]^ adopted, whose accuracy in materials state had been particularly discussed in prior research^[2]^, here we discuss its validation from two aspects:

- 1. Us-Up Hugoniot relation


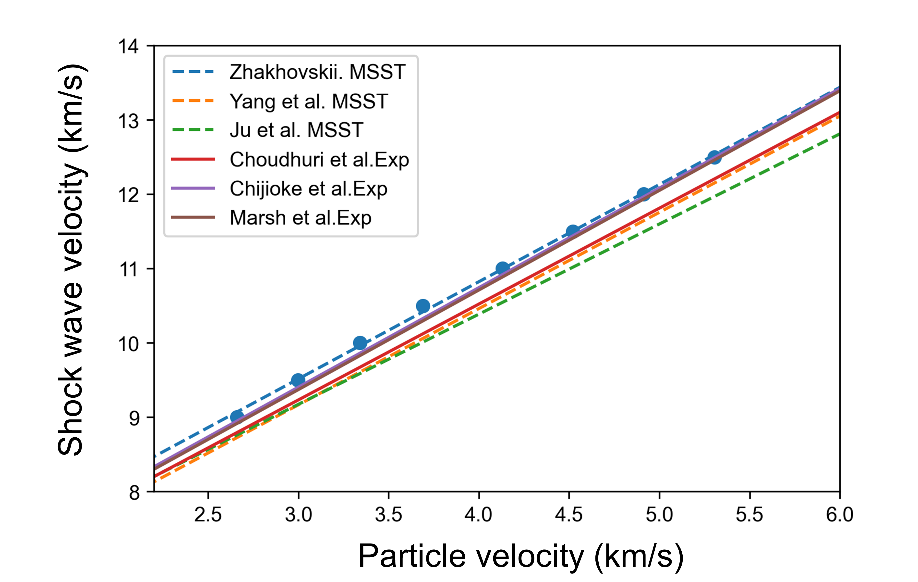


Fig. S 1 Hugoniot data calculated for Al in our pre-simulation

For shock-related simulations, the material’s Hugoniot parameters are crucial for validating the simulation's reasonableness, In our preliminary simulations, the Us-Up relation (shock wave velocity and particle velocity) through MSST of Al match experimental data very well, as shown in Fig. S 1, where the blue discrete points represent our NEMD experimental data.

- 1. Elastic modulus

Since this simulation involves the propagation of elastic precursor, it is necessary to verify the accuracy of the potential function in describing the elastic behavior of the material. The stress-strain curves obtained from NPT tensile simulations (at a strain rate on the order of 10⁹) show that the Young’s modulus of single-crystal aluminum with different orientations is 75 Gpa for [100] and 90 GPa for [111], which is higher, due to the ideal FCC structure, but acceptable compared to experimental values (70GPa).

In summary, we consider the adopted potential function to be sufficiently accurate in this study.


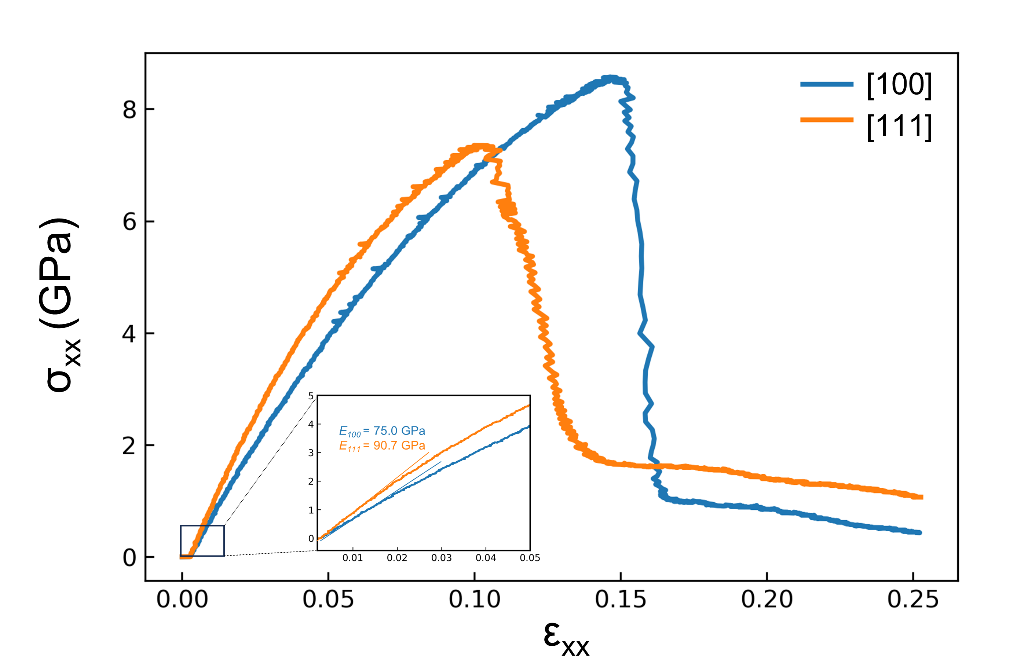


Fig. S 2 Stress-strain curve in NPT tensile simulation

The length of boxes in shock direction (x-) is kept near to 90 nm with 30nm in y- and z- direction. Free boundary conditions are applied in x-direction, while periodic boundary conditions are applied in y- and z- to reduce the boundary effect. Energy minimization is performed by the conjugate gradient (CG) method to reach a local minimum-energy state, then, samples are relaxed in an NPT ensemble at 300 K and 0 Pa for 50 ps to enable appropriate atomic relaxations at the grain boundaries. After that, shock simulation is carried out in an NVE ensemble with a timestep of 1 fs.

We use rigid piston method to introduce shock wave^[3]^, a 5 Å-thick rigid piston on the left side of the target moves along x- direction at 1.3 km/s for a period to bring spall fracture in all sample meanwhile avoiding intense thermal soften or micro-spall. Pulse duration holds constant at 10 ps such that spall appears predictably in the middle of the sample. After loading’s completion, the rigid piston is removed and subsequent rarefaction wave generates and propagates.

We snapshot the atomic configuration with the powerful software Open Visualization Tool (OVITO)^[4]^, Moreover, integrated modules Construct Surface Mesh are used to identify voids or damage accumulations.

Based on binning analysis, the maximum tensile strength is treated as the spall strength, $\sigma_{\mathrm{sp}}$, we do not calculate $\sigma_{\mathrm{sp}}$ by sound approximation method due to complex wave interaction appear with different orientation. Tensile stress was calculated from the Virial- theorem component on the x-axis of all atoms. We note that the thermal vibration velocity in the x direction within the non-equilibrium region behind the wavefront cannot be directly obtained. Yet, it is approximated by v_ix_ = 1/2 (v_iy_+v_iz_) due to the isotropy of thermal vibration^[5]^.

1. **Orientation setup**

We choose 8 representative FCC orientations, which are distributed uniformly in inverse-pole graph, as shown in Table.1 below:

Table 1 Orientation of single crystal Al model

| Orientation | 1 | 2 | 3 | 4 | 5 | 6 | 7 | 8 |
| --- | --- | --- | --- | --- | --- | --- | --- | --- |
| X | 100 | 114 | 112 | 111 | 122 | 123 | 012 | 011 |
| Y | 010 | 1-10 | 20-1 | 1-10 | 20-1 | -210 | 5-21 | -21-1 |
| Z | 001 | 22-1 | 1-52 | 11-2 | 2-54 | 36-5 | 12-1 | 11-1 |

1. **Spall anisotropy under ramp wave loading**

In this paper, the anisotropy of the damage rate is attributed to the separation of the shock wave; however, this result likely contains the influence of the orientation effect of the intrinsic damage susceptibility of single-crystal aluminum with different orientations, and we designed an ingenious simulation test of ramp-wave loading to exclude this interference. Compared to square wave used in main text, ramp-wave loading refers to the gradual elevation of pressure, which is realized in MD by the rising front of the velocity of the piston at a given time, see Fig. S 3(c). Since the ramp wave gradually catches up and evolves into a square wave under supporting load, in contrast to direct square wave loading, ramp wave loading is able to change only the waveform of the wave system propagation without introducing a drastic temperature rise internally^[6, 7]^, while ensuring that the peak values of the loading impulse, pressure, and strain rate are consistent.


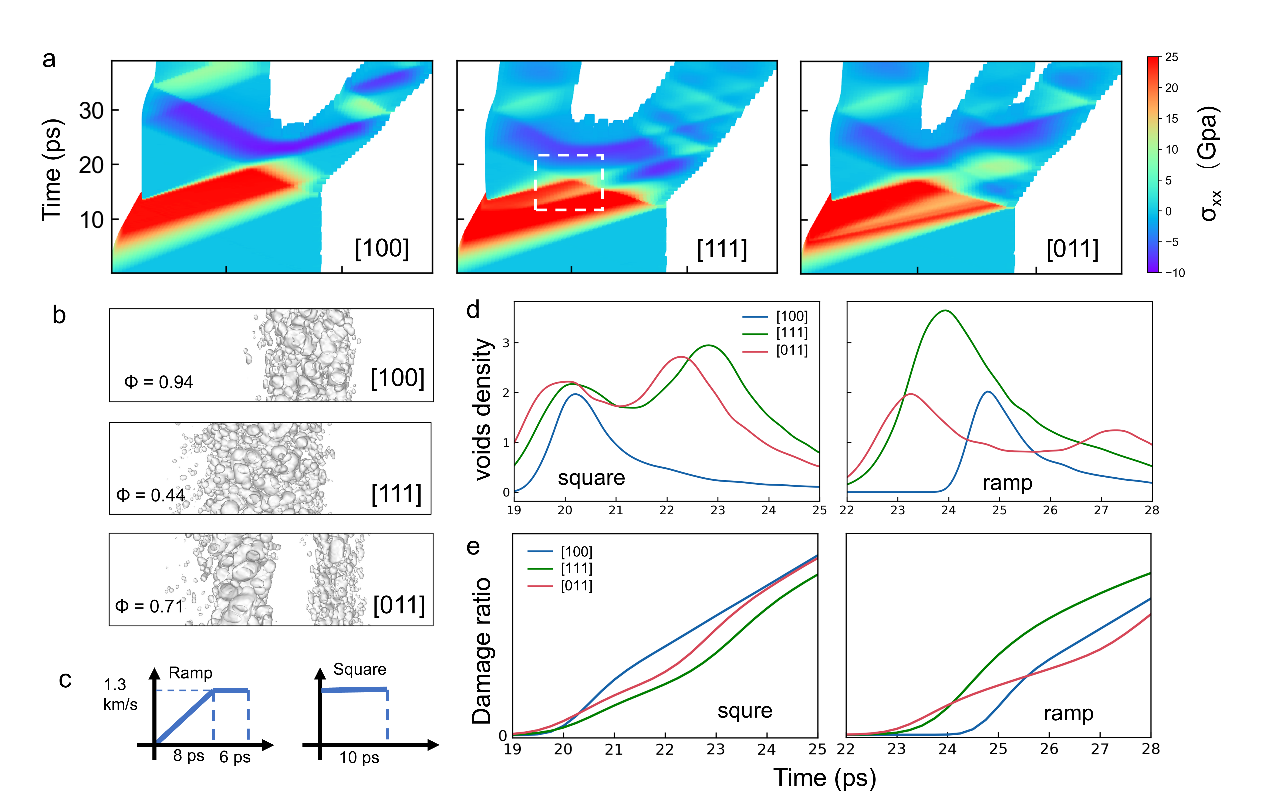


Fig. S 3 (a) Position-Time (X-T) clouds in three main orientations under ramp wave loading. (b) voids distribution at D = 0.1. (c) a comparison of piston’s velocity, (d)voids number and (e) damage ratio history between ramp and square wave.

1. **Modified Nucleation and Growth Model and NSGA-ii algorithm**

The classical nucleation and growth (NAG) model^[8]^ has been widely applied in the field of dynamic failure^[9-11]^. However, its assumption that all voids evolve independently makes it unsuitable for the later stages of failure, where large-scale void coalescence occurs.

In NAG model, the total change in the void volume, $\Delta V$, was the sum of the void nucleation $\Delta V_{n}$ and void growth $\Delta V_{g}$,

$$\Delta V=\Delta V_{n}+\Delta V_{g}$$

Chen et al. further refined this model (Modified NAG model) by incorporating an improved void merging term $\Delta V_{c}$^[12]^.

$$\Delta V=\Delta V_{n}+\Delta V_{g}+\Delta V_{c}$$

Chen assumed for simplicity that the change (reduction) in the number density of voids due to void coalescence was linearly dependent on the current void number density as well as the void radius at the growth stage, In addition, they introduced the concept of the characteristic void size, $R_{t}$, as a measure of the ensemble average void size, at time interval $\Delta t$, the change in $R_{t}$, can be expressed as:

$${\Delta R}_{t}=R_{t}\left( exp\left\lceil\frac{1}{4}\left( \frac{max(\sigma_{m}-\sigma_{g0},0)}{\eta} \right)\Delta t \right\rceil-1 \right)$$

Assuming a critical number density, $k_{V}$, and critical void radius $k_{R}*R_{0}$ beyond which void coalescence took place, the reduction in the void number density due to coalescence is:

$$\Delta N_{Vc}=max(N_{V}-k_{V},0)\frac{max(R_{t}-k_{R}*R_{0},0)}{R_{c}}\frac{\Delta t}{t_{n}}$$

As they assumed spherical voids in this paper, the contribution to the void volume, $\Delta V_{c}$, should be directly related to ${R_{t}}^{3}$ and expressed as:

$$\Delta V_{c}=V_{c0}*{[max(R_{t}-k_{R}*R_{0},0)]}^{3}\Delta t$$

where V_c0_ is the base void coalescence rate (units m^-3^ps^-1^). Hence, the change in the void number density, $\Delta N_{V}$, due to void nucleation and coalescence in the MNAG model is:

$$\Delta N_{V}=\Delta N_{V_{N}}-\Delta N_{V_{C}}$$

Building on Chen’s model, this study models dual spall in [111] sample as two independent single-spall events. We use the polynomial-fitted stress history from MD simulations as input and then the Nondominated Sorting Genetic Algorithm II (NSGA-II)^[13]^ module in the Pymoo package is employed to iteratively determine the optimal parameters in MNAG, achieving a good fitting performance. The optimized parameters are as follows:

Table. S 1 fitted parameters in MNAG model

|  | N_0_  (m^-3^ps^-1^) | σ_n0_ (GPa) | σ_n_  (GPa) | η  (GPa ps) | σ_g0_  (GPa) | R_c_  (m) | V_c0_  (m^-3^ps^-1^) | k_V_  (m^-3^) | k_R_ | k_vs_ |
| --- | --- | --- | --- | --- | --- | --- | --- | --- | --- | --- |
| 100 | 1.95  ×10^22^ | 4.57 | 0.90 | 0.38 | 6.76 | 4.7  ×10^-10^ | 5.95  ×10^25^ | 4.9  ×10^23^ | 1.60 | 1.35 |
| 111 | 1.80  ×10^22^ | 4.08 | 0.95 | 0.36 | 6.6 | 4.7  ×10^-10^ | 3.73  ×10^25^ | 4.9  ×10^23^ | 1.3 | 1.8 |

1. **Effective fracture surface energy calculation**

The total E_ef_ calculation is based on Grady’s model^[14]^ $E_{ef}=YDs$, where Y represents the flow stress of the spall region, D is the damage degree (here we choose D = 0.1), and s denotes the equivalent void size. Assuming all voids to be cubic structures with the same surface area, $s$ can be obtained through the data on void surface area and number at the moment D = 0.1. However, the above equation strongly rely on uniform nucleation assumption, to compare single and dual spallation, we calculate the total fracture surface energy $E_{ef\_tot}=E_{ef}N$, N denotes void number. Despite the simplicity of this model, it has been proven adequate for discussing spall resistance at different sizes from an energetic perspective. The relative data of different samples are attached below.

Table. S 2 Data used in Grady’s model

|  | Y (GPa) | D | s (nm) | N |
| --- | --- | --- | --- | --- |
| 100 | 0.76 | 0.1 | 4.73 | 160 |
| 111 | 1.35 | 0.1 | 2.50 | 1263 |

**Reference**

[1] V.V. Zhakhovskii, N.A. Inogamov, Y.V. Petrov, S.I. Ashitkov, K. Nishihara, Molecular dynamics simulation of femtosecond ablation and spallation with different interatomic potentials, Applied Surface Science 255(24) (2009) 9592-9596.

[2] D. Jiang, J. Shao, B. Wu, P. Wang, A. He, Sudden change of spall strength induced by shock defects based on atomistic simulation of single crystal aluminum, Scripta Materialia 210 (2022) 114474.

[3] B.L. Holian, P.S. Lomdahl, Plasticity Induced by Shock Waves in Nonequilibrium Molecular-Dynamics Simulations, Science 280(5372) (1998) 2085-2088.

[4] A. Stukowski, Visualization and analysis of atomistic simulation data with OVITO–the Open Visualization Tool, Modelling and Simulation in Materials Science and Engineering 18(1) (2010) 015012.

[5] X. Tian, J. Cui, K. Ma, M. Xiang, Shock-induced plasticity and damage in single-crystalline Cu at elevated temperatures by molecular dynamics simulations, International Journal of Heat and Mass Transfer 158 (2020) 120013.

[6] J.M.D. Lane, S.M. Foiles, H. Lim, J.L. Brown, Strain-rate dependence of ramp-wave evolution and strength in tantalum, Physical Review B 94(6) (2016).

[7] W. Li, E.N. Hahn, P.S. Branicio, X. Yao, X. Zhang, B. Feng, T.C. Germann, Rate dependence and anisotropy of SiC response to ramp and wave-free quasi-isentropic compression, International Journal of Plasticity 138 (2021).

[8] L. Seaman, D.R. Curran, D.A. Shockey, Computational models for ductile and brittle fracture, Journal of Applied Physics 47(11) (1976) 4814-4826.

[9] H. Zhang, H. Peng, X. Pei, S. Yao, H. He, P. Li, Critical damage degree model of spall fracture in ductile metals, Journal of Applied Physics 130(12) (2021).

[10] Y. Wang, H. He, L. Wang, Critical Damage Evolution model for spall failure of ductile metals, Mechanics of Materials 56 (2013) 131-141.

[11] X. Yang, X. Zeng, J. Wang, J. Wang, F. Wang, J. Ding, Atomic-scale modeling of the void nucleation, growth, and coalescence in Al at high strain rates, Mechanics of Materials 135 (2019) 98-113.

[12] J. Chen, D.J. Luscher, S.J. Fensin, The Modified Void Nucleation and Growth Model (MNAG) for Damage Evolution in BCC Ta, Applied Sciences 11(8) (2021).

[13] K. Deb, U.B. Rao N, S. Karthik, Dynamic multi-objective optimization and decision-making using modified NSGA-II: a case study on hydro-thermal power scheduling, International conference on evolutionary multi-criterion optimization, Springer, 2007, pp. 803-817.

[14] D.E.Grady, THE SPALL STRENGTH OF CONDENSED MATTER, Journal of the Mechanics and Physics of Solids 36(3) (1988) 353-384.


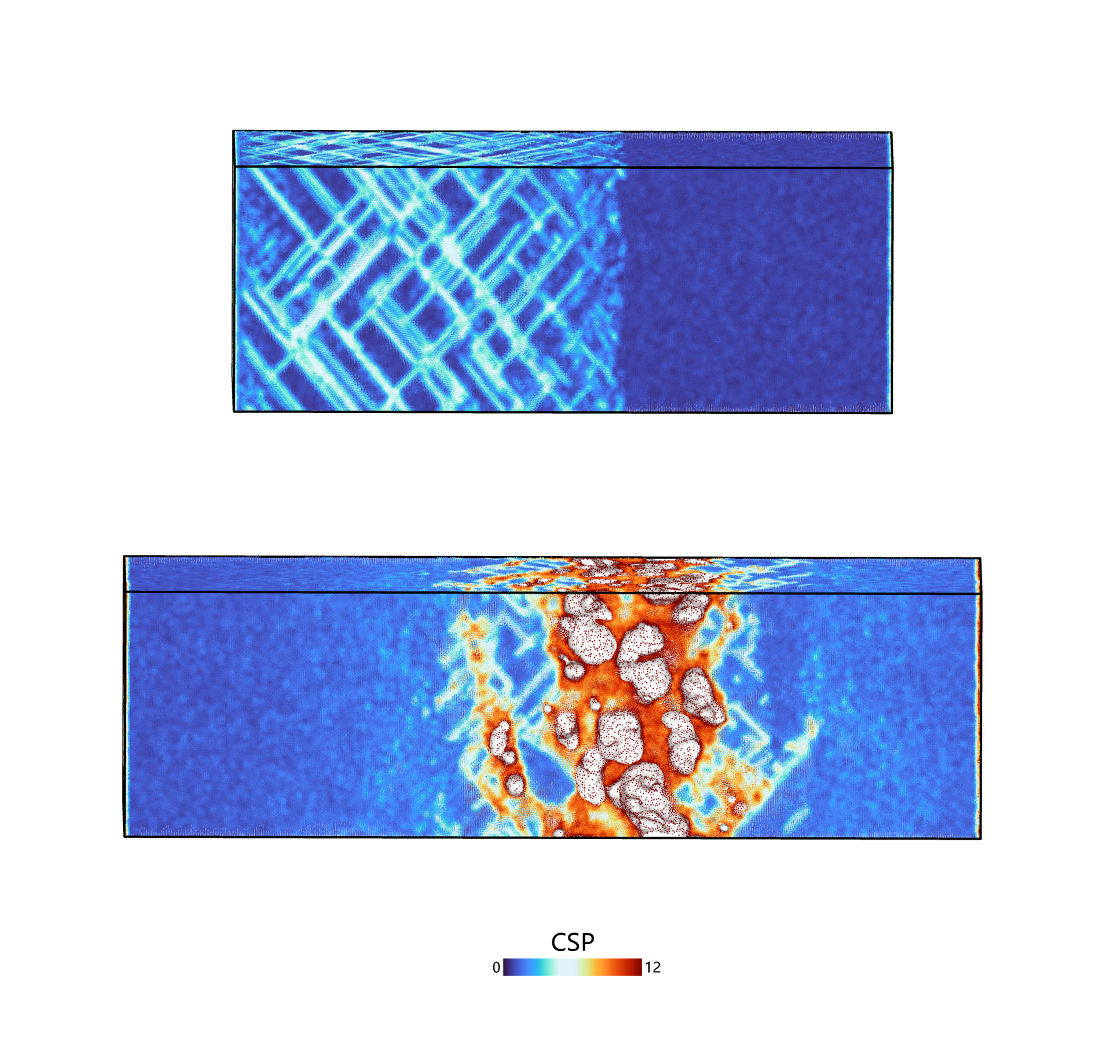


Movie S1 CSP -colored atomic snapshots during shock compression (upper) and single-spall fracture process (lower) of [100] single crystal aluminum, corresponding to Fig. 2(b), the movie was paly back at 4 fps


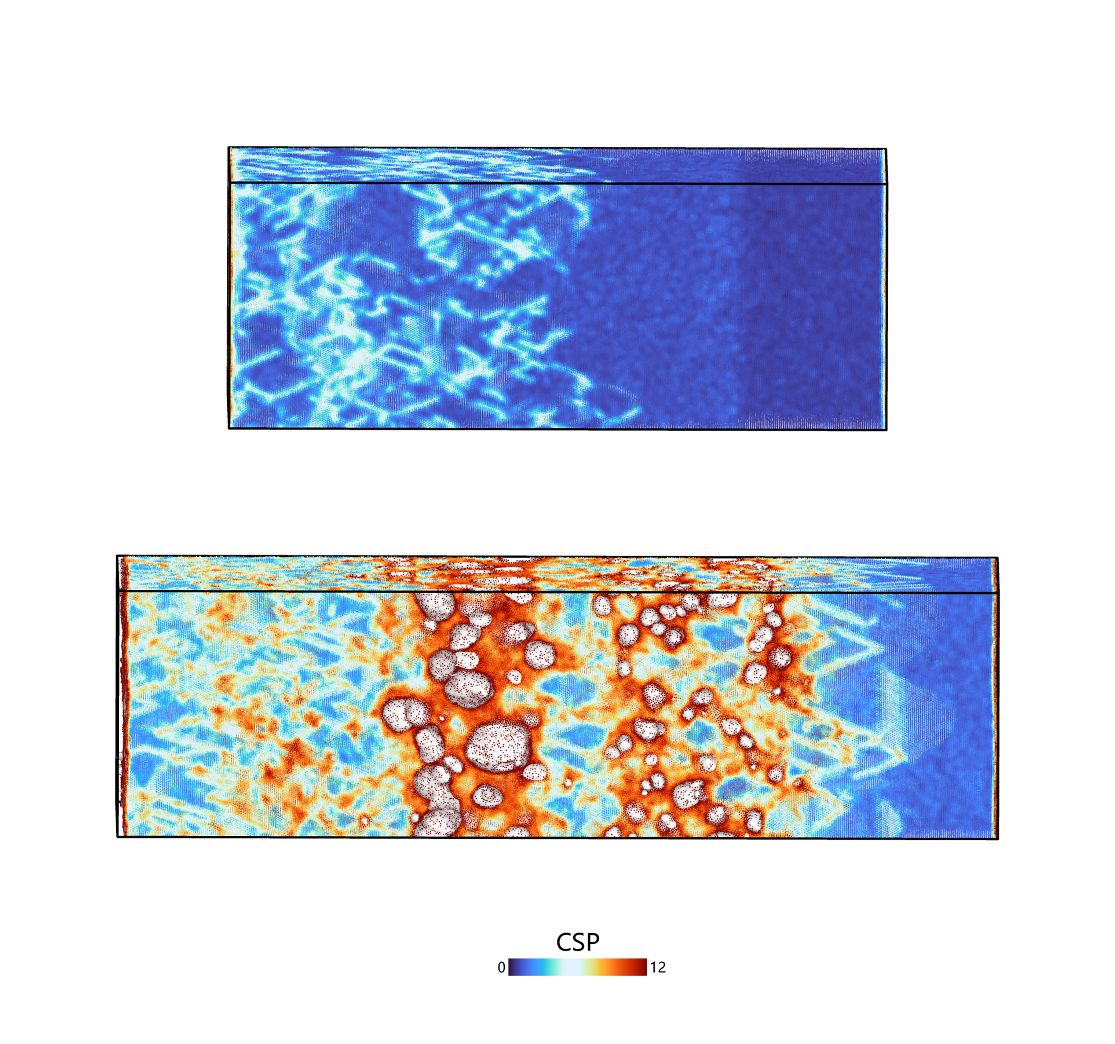


Movie S2 CSP -colored atomic snapshots during shock compression (upper) and dual-spall fracture (lower) process of [111] single crystal aluminum, corresponding to Fig. 2(b), the movie was paly back at 4 fps
